# Quantitative trait loci identification and breeding value estimation of grain weight-related traits based on a new wheat 50K single nucleotide polymorphism array-derived genetic map

**DOI:** 10.3389/fpls.2022.967432

**Published:** 2022-08-30

**Authors:** Xiaofeng Liu, Zhibin Xu, Bo Feng, Qiang Zhou, Guangsi Ji, Shaodan Guo, Simin Liao, Dian Lin, Xiaoli Fan, Tao Wang

**Affiliations:** ^1^Chengdu Institute of Biology, Chinese Academy of Sciences, Chengdu, China; ^2^University of Chinese Academy of Sciences, Beijing, China; ^3^Innovative Academy for Seed Design, Chinese Academy of Sciences, Beijing, China

**Keywords:** wheat, genetic map, QTL analysis, grain weight, breeding value

## Abstract

Mining novel and less utilized thousand grain weight (TGW) related genes are useful for improving wheat yield. In this study, a recombinant inbred line population from a cross between Zhongkemai 138 (ZKM138, high TGW) and Chuanmai 44 (CM44, low TGW) was used to construct a new Wheat 50K SNP array-derived genetic map that spanned 1,936.59 cM and contained 4, 139 markers. Based on this map, ninety-one quantitative trait loci (QTL) were detected for eight grain-related traits in six environments. Among 58 QTLs, whose superior alleles were contributed by ZKM138, *QTgw.cib-6A* was a noticeable major stable QTL and was also highlighted by bulked segregant analysis with RNA sequencing (BSR-Seq). It had a pyramiding effect on TGW enhancement but no significant trade-off effect on grain number per spike or tiller number, with two other QTLs (*QTgw.cib-2A.2* and *QTgw.cib-6D*), possibly explaining the excellent grain performance of ZKM138. After comparison with known loci, *QTgw.cib-6A* was deduced to be a novel locus that differed from nearby *TaGW2* and *TaBT1*. Seven simple sequence repeat (SSR) and thirty-nine kompetitive allele-specific PCR (KASP) markers were finally developed to narrow the candidate interval of *QTgw.cib-6A* to 4.1 Mb. Only six genes in this interval were regarded as the most likely candidate genes. *QTgw.cib-6A* was further validated in different genetic backgrounds and presented 88.6% transmissibility of the ZKM138-genotype and a 16.4% increase of TGW in ZKM138 derivatives. And the geographic pattern of this locus revealed that its superior allele is present in only 6.47% of 433 Chinese modern wheat varieties, indicating its potential contribution to further high-yield breeding.

## Introduction

Bread wheat (*Triticum aestivum* L.) is one of the most important crops worldwide, providing approximately 20% of calories and 25% of protein for humans (FAO, 2017).^1^ The global population is reportedly expected to reach nine billion by 2050, which will require an increase in overall food production of at least 70% to meet future food needs.^2^ Therefore, the steady growth of wheat production is an essential factor in ensuring food security.

Thousand grain weight (TGW), one of three yield components, affects wheat production together with the grain number of spike (GNS) and tiller number (TN). It is a quantitative trait controlled by multiple genes and shows higher heritability than GNS and TN (Simmonds et al., 2014). Previous reports have found that TGW is influenced by multiple grain-related traits (GRTs), including grain size traits [such as grain length (GL), grain width (GW), etc.], and the grain shape traits [such as grain roundness (GR), etc.] (Kumar et al., 2016; Wang et al., 2019a; Guan et al., 2020). These GRTs, especially the grain size traits, are positively correlated with TGW and thus regarded as genetic improvement targets together with TGW in the breeding process (Gegas et al., 2010). Therefore, simultaneously dissecting the novel genetic background controlling TGW and related GRTs can effectively improve wheat yield potential.

Researchers have identified several genes for TGW to facilitate high-yield genetic improvements, such as *TaGW2* (Su et al., 2011), *TaBT1* (Wang et al., 2019b), as well as *TaTPP-6AL* (Zhang et al., 2017). *TaGW2-6A*, an E3 RING ligase gene, is located near the centromere of chromosome 6AS (237.734–237.759 Mb) (Su et al., 2011) and also is one of the most famous and well-studied weight relative genes in common wheat. Previous studies have reported that several single nucleotide polymorphisms (SNPs) or variations could account for yield increases. For instance, SNPs at –593 bp of the promoter region (Hap-6A-A) in Chinese modern varieties (Su et al., 2011) and –494 bp (Hap_5) in Indian wheat (Jaiswal et al., 2015) were discovered, and their corresponding cleaved amplified polymorphic sequence (CAPS) markers were developed for marker-assisted selection(MAS) for improving grain weight. Moreover, the haplotype distributions analysis revealed that the Hap-6A-A was the preferred allele in 265 Chinese mini-core collections and increased TGW (3.11 g). In the 5′ flanking region, Zhai et al. (2018) identified a novel and rare *TaGW2-6A* allele and found that only three accessions among 848 hexaploid, 238 tetraploid, and 27 diploid wheat accessions contained the favorable allele (*TaGW2-A1*) increasing TGW. In addition, two mutations in the coding sequence have also been reported to be associated with TGW and grain size: a frame-shift mutation in exon 8 (Yang et al., 2012; Du et al., 2016) and a splice acceptor site mutation in exon 5 (Simmonds et al., 2016). The markers developed based on these reported loci are important tools for detecting *TaGW2-6A* haplotypes and estimating their effects on TGW.

In addition to the known genes, hundreds of QTLs for TGW were primarily reported overall wheat chromosomes (Cao et al., 2020; Chen et al., 2020b; Hu et al., 2020; Liu et al., 2020; Xin et al., 2020; Li et al., 2022) and a large number of them were co-localized with other GRTs. For example, Li et al. (2022) detected forty-two QTLs controlling TGW, GW, and GL and explaining 3.26–34.06% of phenotypic variation on chromosomes 2A, 3A, 5A, 6A, 3B, and 4B based on the new genetic map from the Wheat 55K SNP array. A major stable QTL for TGW was co-located with that for GW on chromosome 4B, which could increase TGW by 9.64% and was considered a novel locus. In addition, Wang et al. (2019a) reported fourteen QTL clusters for TGW and GRTs based on a Wheat 90K SNP array-derived map, and five of them were likely novel loci and improved the corresponding traits, making them worthy of further exploration and application. Consequently, QTL mapping is an effective method for discovering novel loci and dissecting the genetic basis of multiple complex related traits.

With the advent of draft and complete genome sequences (International Wheat Genome Sequencing, 2014, 2018)^3^, high-density SNP arrays (Wang et al., 2014; Winfield et al., 2016) have greatly accelerated and facilitated wheat genetic studies. To date, several kinds of wheat SNP arrays have been reported, including 9K (Cavanagh et al., 2013), 35K (King et al., 2017), 50K (Song et al., 2021), 55K (Li et al., 2022), 90K (Wang et al., 2019a), 820K (Winfield et al., 2016), and 660K (Cui et al., 2017) SNP arrays. These SNP arrays have contributed to the detection of important genetic traits and excavation of superior genes in wheat. The technology for transformation and validation of the linked SNPs has also been developed (Wang et al., 2019b; Deng et al., 2022), such as KASP, CASP, and InDel markers (Jaiswal et al., 2015; Zhai et al., 2018; Li et al., 2022), gradually facilitating the detection or validation of the target QTLs. For example, Liu et al. (2020) discovered that the favorable allele of *TaFT-D1* had been positively selected during Chinese wheat breeding and increased TGW by 4.33 g by using an effective KASP marker, further indicating these closely linked SNPs can indeed facilitate MAS.

In this study, we developed a recombinant inbred line (RIL) population based on the cross between ZKM138 (high grain weight) and CM44 (low grain weight) with the aims to: (1) construct a genetic map based on the Wheat 50K SNP array to analyze the genetic basis of the superior grain and yield performance of ZKM138; (2) dissect the valuable QTLs controlling TGW and GRTs; (3) develop effective markers to assess the application and distribution of target QTL.

## Materials and methods

### Plant materials and field trials

**FIGURE 1:**
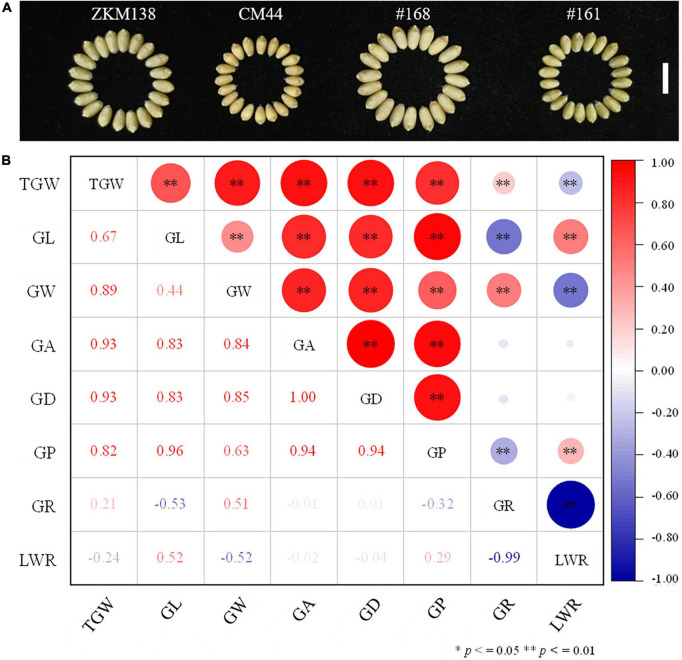
Performance of the measured grain related traits in the BC-RILs population. **(A)** Grain phenotype of the parents ZKM138 and CM44, and two representative lines of the BC-RILs population. The white line represents a scale = 1 cm. **(B)** Correlation analysis between TGW and GRTs in the BC population based on the BLUE dataset. * and ^**^ indicate significance at *P* ≤ 0.05 and *P* ≤ 0.01, respectively.

An F_7–9_ RIL population, including 170 lines from a cross between ZKM138 and CM44 (named BC-RILs), was used for genetic map construction and QTL mapping. ZKM138 is a high-yield wheat variety with excellent TGW and grain performance. It has been consecutively listed as a leading variety in the Chengdu Plain since it was released in 2015 by the Chengdu Institute of Biology, CAS (CIBCAS) (Figure 1A). CM44, released in 2004, is also a representative variety in Sichuan Province with good quality, but its grain weight and size are obviously smaller than those of ZKM138. Another RIL population, derived from a cross between the public parent ZKM138 and a variety with smaller grain (CD1437) (ZC), containing 152 lines, was employed to validate the target QTL in different genetic backgrounds. Additionally, seventy-nine derivatives of ZKM138 also developed by CIBCAS were used to validate the target QTL further and assess its application potential in our actual breeding process. In addition, 433 Chinese varieties and 191 wheat varieties from other countries were employed to evaluate the distribution of the target locus and its potential application value for future TGW improvement.

The RILs and parents were evaluated in two field experiments in Shifang (SF, 104°11′E, 31°6′N) and Shuangliu (SL, 103°52′E, 30°34′N), in 2019, 2020, and 2021 planted in a randomized block design. Each field trial was planted with two blocks. Each block contained the complete population lines and parents. The organic fertilizer (300 kg ha^–1^), nitrogen fertilizer (120 kg ha^–1^), phosphate fertilizer (50 kg ha^–1^), and potash fertilizer (50 kg ha^–1^) as a base fertilizer were applied only before sowing. The soil nutrition conditions in 0–50 cm soil depth were nitrogen (N) content 1.30 g kg^–1^, phosphate (P) content 820.07 mg kg^–1^, potash (K) content 29.28 g kg^–1^, ammonium nitrogen content 11.611 mg kg^–1^, and nitrate-nitrogen content 8.89 mg kg^–1^ (Shuangliu) and N content 1.128 g kg^–1^, P content 772.33 mg kg^–1^, K content 19.51 g kg^–1^, ammonium nitrogen content 42.75 mg kg^–1^, and nitrate-nitrogen content 87.35 mg kg^–1^ (Shifang), respectively.

The field trials were carried out for the ZC population in four crop seasons of 2016–2018 at Shifang (E1, E3, and E5) and three crop seasons of 2016–2018 (E2, E4, and E6) at Shuangliu. Moreover, seventy-nine derivatives of ZKM138 were planted and evaluated at Shuangliu in 2019–2020.

Each line was single-seed planted in double-row plot with a length of 1 m, 20 seeds per row, and a row spacing of 0.25 m. Field management and disease control were performed according to the common practices for wheat production.

### Phenotypic evaluation and statistical analysis

At maturity, at least six representative samples planted in the middle of each line were harvested and evaluated for the following eight traits using SC-G software (Wseen, Hangzhou, Zhejiang, China): TGW (Thousand Grain Weight), GL (Grain Length), GW (Grain Width), GA (Grain Area), GD (Grain Diameter), GP (Grain Perimeter), GR (Grain Roundness), and LWR (Length to Width Ratio) (Kumar et al., 2016).

Basic statistical analyses, frequency distribution, and correlation coefficients among traits were conducted using SPSS version 25.0 for Windows (IBM SPSS, Armonk, NY, United States). The best linear unbiased estimator (BLUE) was calculated using QTL IciMapping software. Broad-sense heritability (*H*^2^) was estimated according to the method described by Smith et al. (1998) and Muqaddasi et al. (2019).

### Bulked segregant analysis with RNA sequencing

At the 15 days after pollination (DPA) stage, the grains of two extreme pools from 20 different lines from BC RILs planted in 2020 in the Shifang experimental field were sampled to construct opposite bulk pairs for RNA sequencing. Total RNA was isolated according to the TRIzol protocol (Invitrogen, Carlsbad, CA, United States). The RNA libraries were sequenced on the Illumina sequencing platform and further analyzed by Genedenovo Biotechnology Co., Ltd. (Guangzhou, China).

### Construction of the genetic map

The genomic DNA of parents and BC populations was extracted from fresh seedling leaves using the CTAB method and hybridized on the Wheat 50K SNP array by CapitalBio Technology (Beijing, China).

IciMapping 4.1 (Meng et al., 2015) and JoinMap 4.1 were used for genetic construction. First, all markers were analyzed using the BIN function of IciMapping 4.1^4^ based on their parameters of “Missing Rate” and “Distortion Value” being set as 20% and 0.001, respectively. Only one marker from each bin (bin marker) was randomly selected to construct the genetic map. Then, the function of ‘Population’ in JoinMap 4.1 was used to create groups with a limit of detection (LOD) score values ranging from 2 to 10. Last, the Kosambi mapping function was used to order the bin markers, with the parameters being set as LOD ≥ 7 and round = 3, in JoinMap 4.1.

### Detection of quantitative trait loci

Quantitative trait loci detection in seven datasets (six environments and the BLUE dataset) of BC-RILs was performed by IciMapping 4.1 with the inclusive composite interval mapping (ICIM). Missing phenotype was deleted in the QTL analysis. Single-environment QTLs were detected using the biparental population (BIP) module with a walking step = 0.001 cM and PIN = 0.001, and a test with 1,000 permutations was used to identify the LOD threshold. A LOD value = 2.5 was used to detect putative QTLs. Moreover, a QTL with a LOD value ≥ 3.5 and phenotypic variation > 10% (on average) that was detected in more than two datasets was considered a major stable QTL (Li et al., 2022). If the confidence intervals of corresponding QTLs overlapped, these QTLs were considered and named one QTL.

Besides, except for single-environment QTL detection based on ICIM, multi-environment QTL analysis was conducted by IciMapping 4.1 using the multi-environment trials (MET) with pre-adjusted parameters: Step = 1 cM, PIN = 0.001, and LOD = 2.5. A QTL was considered significantly related to the environment when LOD (AbyE) > 3. QTLs were named according to the International Rules of Genetic Nomenclature, where “cib” represents Chengdu Institute of Biology.

### Association of the known genes

Sixteen functional KASP markers related to 14 reported yield-related genes, including *TaSus2-2A*, *TaCwi*, *TaGS*, *TaGS2-B1*, *TaSus2*, *GS5*, *TaTGW6*, *TaCWI*, *TaGW2*, *TaSus1-7A*, *TaTGW-7A*, *TaMOS1-7A*, *TaSus1-7B*, and *TaGS-D1*, were used to detect polymorphisms in parents by China Golden Marker in Beijing.

The physical interval of the target QTL overlapped with *TaGW2* (Su et al., 2011) and was adjacent to *TaBT1* (Wang et al., 2019b). Thus four markers were used to detect*TaGW2* in the parents and lines, including two markers (Su et al., 2011; Simmonds et al., 2016) in the coding region and two markers (Su et al., 2011; Zhai et al., 2018) in the promoter region. Sequence analysis of the promoter region and coding region of *TaGW2* was also performed. Meanwhile, two PCR markers listed in Supplementary Table 1 were used to detect *TaBT1*.

### Development of kompetitive allele-specific polymerase chain reaction markers and simple sequence repeat markers

Kompetitive allele-specific polymerase chain reaction markers and SSR markers (Supplementary Table 1) were developed to construct the saturated genetic map of target QTLs based on information from the Wheat 50K SNP array and BSR-Seq in the preliminary QTL interval. According to the previous report (Li et al., 2022), the reaction conditions and systems of the polymerase chain reaction (PCR) system and the KASP arrays were conducted.

### Prediction of candidate genes

Genes within the target region were extracted from the re-sequencing data of the parents. Functional annotation was performed by http://www.uniprot.org/. The gene expression profiles were obtained from the Wheat Expression Browser.^5^ Graphs of expression values were drawn in TBtools software (v.1.098) (Chen et al., 2020a).

## Results

### Phenotypic evaluation and correlation analysis

The phenotypic performance of eight traits (TGW, GL, GW, GA, GD, GP, GR, and LWR) in two parents and the BC-RILs across seven datasets are shown in Table 1. Compared with CM44, ZKM138 had significantly higher TGW and five grain size traits, including GL, GW, GA, GD, and GP (Figure 1A), while the grain shape traits (GR and LWR) were not significantly different between the two parents. In the BC population, the absolute values of skewness and kurtosis were almost <1 (Table 1), indicating that the phenotypic data were approximately normally distributed in this population. Strong transgressive segregation exceeding the limits of both parents was observed, indicating that favorable alleles were distributed by the two parents (Table 1). Broad sense heritability (*H*^2^) was greater than 0.80 across each environment for all traits.

**TABLE 1:** Phenotypic variation and heritability (*H*^2^) of thousand grain weight (TGW) and grain-related traits (GRTs), for the parents and the BC-RILs in different environments.

Traits	Env.	Parents		BC-RILs							
		ZKM138	CM44	Min	Max	Mean	SD	CV	Sk.	Ku.	
TGW (g)	1E	46.62	24.54***	22.11	59.36	41.79	7.12	17.03%	–0.07	–0.17	0.96
	2E	57.79	39.67***	31.79	68.44	52.07	6.13	11.77%	–0.14	0.29	
	3E	56.66	39.22***	37.61	73.01	54.17	6.07	11.21%	0.24	0.62	
	4E	58.56	42.80***	29.47	67.79	49.72	7.20	14.47%	–0.32	0.56	
	5E	55.35	38.28***	31.50	67.34	50.06	5.93	11.85%	–0.10	0.20	
	6E	60.14	43.08***	36.16	68.28	51.78	5.83	11.25%	–0.04	0.07	
	BLUE	57.40	34.85	34.80	64.62	49.93	5.48	10.97%	–0.14	0.18	
GL (mm)	1E	6.23	6.09***	5.13	7.25	6.28	0.34	5.38%	–0.09	0.79	0.91
	2E	5.88	5.01***	5.16	7.19	5.99	0.37	6.22%	0.72	0.89	
	3E	6.02	5.37***	5.12	7.05	6.14	0.32	5.26%	0.04	0.50	
	4E	6.01	5.25***	5.02	6.84	5.91	0.31	5.22%	–0.07	0.36	
	5E	6.10	5.46***	5.56	7.06	6.28	0.29	4.68%	0.04	–0.05	
	6E	6.26	5.29***	5.30	6.91	6.06	0.31	5.14%	0.17	0.30	
	BLUE	6.06	5.13	5.27	6.79	6.11	0.29	4.77%	–0.15	0.21	
GW (mm)	1E	2.94	2.95***	2.22	3.37	2.87	0.21	7.49%	–0.09	–0.17	0.87
	2E	2.97	2.61***	2.43	3.41	2.89	0.16	5.69%	0.31	0.61	
	3E	3.09	2.69***	2.54	3.40	2.98	0.16	5.46%	–0.14	0.13	
	4E	3.14	2.86***	2.40	3.39	2.99	0.21	6.89%	–0.64	0.07	
	5E	3.06	2.69***	2.53	3.27	2.91	0.14	4.73%	–0.14	–0.19	
	6E	3.31	2.94***	2.60	3.41	3.09	0.14	4.60%	–0.56	0.48	
	BLUE	3.13	2.71	2.55	3.30	2.96	0.14	4.64%	–0.30	0.09	
GA (mm^2^)	1E	14.29	13.82***	8.48	19.84	14.32	1.73	12.07%	0.06	0.81	0.83
	2E	14.14	10.59***	10.85	18.72	13.82	1.53	11.04%	0.87	0.96	
	3E	14.99	11.47***	11.15	19.99	14.97	1.58	10.55%	0.42	0.86	
	4E	15.24	12.01***	9.45	19.54	14.54	1.74	11.93%	0.21	0.90	
	5E	15.12	11.77***	11.17	18.24	14.59	1.18	8.11%	–0.03	0.14	
	6E	16.60	12.47***	11.11	18.74	14.96	1.28	8.56%	–0.07	0.44	
	BLUE	14.97	10.91	11.39	18.12	14.56	1.19	8.17%	–0.09	0.34	
GD (mm)	1E	4.24	4.18***	3.53	4.97	4.24	0.25	5.82%	0.05	0.39	0.85
	2E	4.22	3.65***	3.70	4.92	4.17	0.22	5.36%	0.62	0.59	
	3E	4.33	3.79***	3.76	4.97	4.34	0.21	4.74%	0.23	0.70	
	4E	4.37	3.90***	3.58	4.93	4.27	0.25	5.84%	–0.03	0.67	
	5E	4.33	3.83***	3.74	4.79	4.27	0.17	4.09%	–0.16	0.18	
	6E	4.57	3.96***	3.74	4.87	4.33	0.19	4.34%	–0.23	0.58	
	BLUE	4.34	3.72	3.82	4.75	4.27	0.17	3.97%	–0.24	0.37	
GP (mm)	1E	15.74	15.33***	13.42	18.41	15.85	0.82	5.15%	0.14	0.55	0.87
	2E	15.36	12.97***	13.38	17.92	15.26	0.84	5.48%	0.64	0.87	
	3E	15.71	13.72***	13.57	17.82	15.58	0.76	4.85%	0.03	0.38	
	4E	15.76	13.71***	12.82	17.77	15.36	0.86	5.61%	0.06	0.52	
	5E	15.63	13.88***	13.81	17.62	15.76	0.67	4.28%	–0.09	0.03	
	6E	16.23	13.84***	13.49	17.61	15.52	0.73	4.69%	0.00	0.36	
	BLUE	15.63	12.90	13.86	17.20	15.56	0.66	4.24%	–0.17	0.18	
GR	1E	0.47	0.49	0.38	0.53	0.46	0.03	6.44%	–0.30	–0.27	0.96
	2E	0.51	0.52	0.41	0.57	0.49	0.03	5.50%	–0.25	–0.01	
	3E	0.51	0.50	0.43	0.55	0.49	0.02	5.09%	0.13	–0.31	
	4E	0.52	0.54	0.41	0.59	0.51	0.03	6.53%	–0.12	–0.15	
	5E	0.50	0.49	0.39	0.53	0.46	0.02	5.11%	–0.03	0.28	
	6E	0.53	0.55	0.46	0.59	0.51	0.02	4.54%	0.17	0.25	
	BLUE	0.52	0.53	0.42	0.56	0.49	0.03	5.31%	–0.15	–0.16	
LWR	1E	2.15	2.08	1.93	2.72	2.22	0.15	6.68%	0.66	0.41	0.95
	2E	1.99	1.94	1.81	2.43	2.07	0.12	5.68%	0.62	0.32	
	3E	1.97	2.03	1.83	2.34	2.08	0.11	5.25%	0.10	–0.45	
	4E	1.92	1.86	1.73	2.48	2.01	0.14	6.97%	0.52	0.18	
	5E	2.04	2.07	1.93	2.61	2.20	0.11	5.20%	0.38	0.69	
	6E	1.91	1.83	1.71	2.23	1.98	0.09	4.57%	0.17	0.03	
	BLUE	1.94	1.89	1.82	2.44	2.09	0.11	5.41%	0.34	0.00	

BLUE, best linear unbiased estimator; Env, environment; SD, standard deviation; CV, coefficient of variation; Sk., skewness; Ku., Kurtosis; H^2^, broad-sense heritability; *** represents significance at P < 0.001.

Notably, the correlation analysis (Figure 1B) revealed that TGW was positively correlated with all grain size traits, such as GL, GW, GA, GD, and GP, especially GA and GD, with the highest correlation coefficient being 0.93 in both cases. For GW and GL, the correlation coefficient of TGW-GW was higher (*r* = 0.89) than TGW-GL (*r* = 0.67), indicating that there might be a more common genetic basis of TGW with GW than GL in BC-RILs. On the other hand, the correlation of TGW with two grain shape traits (GR and LWR) was relatively weak (*r* = 0.21/–0.24).

### New genetic map based on the wheat 50K single nucleotide polymorphism array for BC-RILs

The scores for the probes were classified into six categories by Affymetrix software: (1) Poly High Resolution (PHR) (16724; 25.02%); (2) No Minor Homozygote (NMH) (25307; 37.86%); (3) Off-target Variant (OTV) (1888; 2.82%); (4) Mono High Resolution (MHR) (3306; 4.95%); (5) Call Rate Below Threshold (CRBT) (1,361; 2.04%); and (6) Other (18,249; 27.3%) (Supplementary Figure 1). Of the 66,834 markers called from the Wheat 50K SNP array, 7,725 (11.56%) markers were polymorphic between the two parents. After removing SNPs with more than 20% missing data or a segregation distortion test *P* < 0.001, 4,139 markers were used for map construction and linkage analysis. These markers were divided into 948 bins. Only one marker was selected from each bin. In total, 467 bins contained only one marker, and the largest bin contained 110 markers on chromosome 1A. Finally, a novel genetic map consisting of 24 linkage groups was mapped on 21 chromosomes of wheat. Chromosomes 3A, 5A, and 6D contained two linkage groups (Supplementary Figure 2 and Supplementary Table 2). Based on the genetic information, all SNP markers, including bin markers and redundant markers, were integrated into the genetic map with a total length of 1,939.78 cM and average interval distance of 2.04 cM per bin and 0.47 cM per marker (Supplementary Table 3). The mapped markers were located in the A (28.78%), B (42.67%), and D (28.56%) genomes with lengths of 596.68, 730.44, and 612.66 cM, respectively. In addition, the lengths of the constructed linkage maps ranged from 10.66 (6D2) to 131.21 cM (7D); the markers on linkage maps ranged from 21 (3A2) to 396 (7B); the average interval distance between adjacent markers ranges from 0.08 (6D2) to 2.11 (5D); and the average interval distance between adjacent bin markers ranged from 1.18 (6D2) to 4.22 cM (1D).

Based on the physical locations of these SNPs in the reference genome (IWGSC RefSeq v1.1), 4,019 markers showed best hits to the CS contigs, and 120 markers (2.90%) were mapped to their homologous and non-homologous linkage groups (Supplementary Figure 3). Marker order was relatively consistent with that of the wheat genome assembly on most chromosomes (Supplementary Figure 3).

### Quantitative trait loci mapping for thousand grain weight and grain-related traits based on the biparental population module

For single environment QTL detection, ninety-one QTLs were identified for eight traits in seven datasets with phenotypic variations ranging from 3.10 to 18.04% and LOD values of 2.50–11.85. And forty-five, fifteen, and thirty-one QTLs were distributed on A, B, and D genomes, respectively; the favorable alleles of fifty-eight (63.74%) QTLs were donated from ZKM138. Thirty-six QTLs were stable QTLs that could be detected in more than two datasets and are presented in detail below, and the remaining fifty-five QTLs were detected in only one dataset (Supplementary Table 4).

For TGW, five stable QTLs were detected on chromosomes 2A, 6A, and 6D1. Among these QTLs, a major stable QTL, *QTgw.cib-6A*, was detected in all datasets, explaining 6.84–14.84% of phenotypic variation. The other QTLs, *QTgw.cib-2A.1*, *QTgw.cib-2A.2*, *QTgw.cib-2A.3*, and *QTgw.cib-6D*, explained 6.90–9.43, 9.38–12.74, 5.96–8.33, and 7.93–8.53%, respectively, of phenotypic variation. Interestingly, the positive alleles of all five loci were contributed by ZKM138, the parent with better grain performance.

For GL, eight stable QTLs were identified on chromosomes 2A, 4A, 5A1, 6A, and 6D1. Two major stable QTLs, *QGl.cib-2A.1* and *QGl.cib-6A*, explained 6.99–14.76 and 7.84–12.80%, respectively, of phenotypic variation. *QGl.cib-4A* explained 4.76-7.77% of phenotypic variation. Five QTLs, *QGl.cib-2A.2*, *QGl.cib-2A.3*, *QGl.cib-5A1*, *QGl.cib-6D1.1*, and *QGl.cib-6D1.2*, explained 3.70–4.30, 7.47–9.81, 4.90–6.75, 4.09–4.49, and 7.34–10.26%, respectively, of phenotypic variation. The positive alleles at seven loci (except *QGl.cib-2A.1*) were contributed by ZKM138.

For GW, three stable QTLs were identified on chromosomes 2A, 6A, and 6D1. *QGw.cib-2A.1*, *QGw.cib-6A*, and *QGw.cib-6D1* explained 8.78–9.08, 6.41–9.61, and 7.13–10.18% of phenotypic variation, respectively. Similarly, ZKM138 contributed to the beneficial alleles at all QTLs.

For GA, three stable QTLs were detected on chromosomes 2A, 6A, and 6D1. *QGa.cib-6A*, a major stable QTL, explained 8.14–14.34% of phenotypic variation. The other QTLs *QGa.cib-2A* and *QGa.cib-6D1* explained 7.89–8.12% and 6.61–7.11% of phenotypic variation, respectively. The superior alleles of the three QTLs were also obtained from ZKM138.

For GD, four stable QTLs were identified on chromosomes 2A, 6A, and 6D1. A major stable QTL, *QGd.cib-6A*, explained 8.91–14.21% of phenotypic variation. *QGd.cib-2A.1*, *QGd.cib-2A.2*, and *QGd.cib-6D1* explained 8.57–8.59, 8.33–8.91, and 6.94–6.95%, respectively, of phenotypic variation. Additionally, the beneficial alleles of these QTLs were all from ZKM138.

For GP, seven stable QTLs were detected on chromosomes 2A, 5A1, 6A, and 6D1. A major stable QTL, *QGp.cib-6A*, explained 8.83–15.51% of phenotypic variation. The remaining QTLs, *QGp.cib-2A.1*, *QGp.cib-2A.2*, *QGp.cib-2A.3*, *QGp.cib-5A*, *QGp.cib-6D.1*, and *QGp.cib-6D.2*, explained 6.66–9.42, 9.02–10.10, 3.10–7.62, 7.26–7.60, 4.96–7.06, and 7.04–12.21%, respectively, of phenotypic variation. The positive alleles of these QTLs (except *QGp.cib-2A.1*) were contributed by ZKM138.

For GR, three stable QTLs were detected on chromosomes 2A, 4A, and 5A1. A major stable QTL, *QGr.cib-2A*, was detected in four datasets, explaining 8.11–17.12% of phenotypic variation. *QGr.cib-4A* and *QGr.cib-5A1* explained 5.14–7.52% and 4.48–10.35% of phenotypic variation, respectively. The superior allele increasing GR at *QGr.cib-2A* was contributed by ZKM138, while CM44 contributed to the positive alleles of *QGr.cib-4A* and *QGr.cib-5A1*.

For LWR, three stable QTLs were identified on chromosomes 2A, 4A, and 5A1. *QLwr.cib-2A* was a major stable QTL that explained 8.11–17.12% of phenotypic variation. *QLwr.cib-4A* and *QLwr.cib-5A1* explained 4.71–8.60 and 4.98–9.40% of phenotypic variation, respectively. The positive alleles of *QLwr.cib-4A* and *QLwr.cib-5A1* were donated by ZKM138, while CM44 contributed to the favorable alleles of QLwr.cib-2A. Consistent with expectations, these results were contrary to QTLs for GR.

### Quantitative trait loci clusters for thousand grain weight and grain-related traits

**FIGURE 2:**
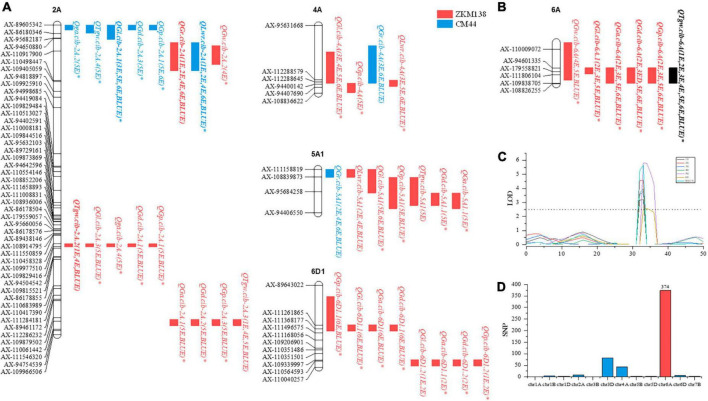
The highlighted QTL regions for TGW and GRTs. **(A)** Summary of QTL clusters detected in the BC-RILs (except C6A). **(B)** The target QTL cluster on chromosome 6A (C6A). **(C)** The LOD value of the major stable QTL (*QTgw.cib-6A*) for TGW in the BC population. The dotted line represents a logarithm of the odds (LOD) = 2.5. **(D)** Identification of candidate SNPs using BSR-Seq. Δ (SNP-index) > 99%.

Based on the stable QTLs detected in a single environment (Supplementary Table 4), eight QTL clusters containing nine major stable QTLs for different traits were highlighted (Table 2). These clustered QTLs shared confidence intervals and thus were inactive of potential pleiotropic effect on the corresponding traits. They were mainly mapped on 2A (three clusters), 4A (one cluster), 5A1 (one cluster), 6A (one cluster), and 6D1 (two clusters), respectively (Figure 2). Notably, the superior alleles on C2A.2, C2A.3, C6A, C6D1.1, and C6D1.2 were all from ZKM138, which increased the corresponding traits.

**TABLE 2:** Quantitative trait loci (QTL) clusters simultaneously affecting thousand grain weight (TGW) and grain-related traits (GRTs) in the BC-RILs.

Cluster	Chr.	No. of QTLs	Genetic interval (cM)	Physical interval (Mb)	QTL (Additive effect, environments)^a^	Coincident genes	Reported QTLs for grain related traits
C2A.1	2A	8	0–15.5	1.37–35.78	TGW (–, 1)*, GL (–, 4)*, GW (+, 1)*, GA (–, 1)*, GD (–, 1)*, GP (–, 2)*, **GR (+, 5)**, LWR (–, 5)*		TGW (Mohler et al., 2016; Su et al., 2018; Liu et al., 2019)
C2A.2	2A	5	69.5–70.5	702.94–729.20	**TGW (+, 3)**, GL (+, 2)*, GA (+, 1)*, GD (+, 2)*, GP (+, 2)*		TGW (Cui et al., 2014; Guan et al., 2018); KNS (Yao et al., 2009; Lee et al., 2014); SN (Lee et al., 2014); GL (Li et al., 2022)
C2A.3	2A	4	93.5–95.5	718.58–755.79	TGW (+, 4), GA (+, 2)*, GD (+, 2)*, GP (+, 2)*	*TaFlo2-A1* (Sajjad et al., 2017)	TGW (Ma et al., 2018; Sukumaran et al., 2018; Liu et al., 2019)
C4A	4A	4	17.5–32.5	16.97–583.95	GL (+, 5)*, GP (+, 1)*, GR (–, 3), LWR (+, 4)*	*TaTGW6* (Hu et al., 2016); *Rht-A1* (Peng et al., 1999); *TB-A1* (Dixon et al., 2018)	TGW (Fatiukha et al., 2020); GNS (Liu et al., 2014; Würschum et al., 2018); GL (Li et al., 2022)
C5A1	5A1	7	0–12.5	0.64–3.65	TGW (+, 1), GL (+, 3)*, GA (+, 1)*, GD (+, 1)*, GP (+, 2)*, GR (–, 4), LWR (+, 3)*		GL (Sun et al., 2017); SN (Mohler et al., 2016);
C6A	6A	6	23.5–36.5	213.15–309.87	TGW (+, 7)*, GL (+, 4)*, GW (+, 3)*, GA (+, 5)*, GD (+, 5)*, GP (+, 5)*	*TaGW2-6A* (Su et al., 2011)	TGW (Li et al., 2022; Sun et al., 2017; Sukumaran et al., 2018; Zhai et al., 2018)
C6D.1	6D1	4	7.5–18.5	67.60–299.20	GL (+, 2)*, GA (+, 2)*, GD (+, 2)*, GP (+, 2)*		TGW (Chen et al., 2019; Liu et al., 2019); GL (Chen et al., 2019); GW (Chen et al., 2019)
C6D.2	6D1	4	27.5–29.5	348.68–411.50	GL (+, 2), GA (+, 1)*, GD (+, 1)*, GP (+, 2)*	*TaGS1a* (Guo et al., 2013)	

^a^Stable and major QTLs are in BOLD typeface; * indicates that the QTL had a significant interaction effect with the environment; + and – indicate that the superior alleles are derived from ZKM138 and CM44, respectively.

C6A contained a major stable QTL for TGW (*QTgw.cib-6A*), which was detected in all seven datasets and was simultaneously clustered with the other four major stable QTLs (*QGl.cib-6A*, *QGa.cib-6A*, *QGd.cib-6A*, and *QGp.cib-6A*) and one other stable QTL (*QGw.cib-6A*), with the ZKM138-derived alleles increasing all these traits.

C2A.2 contained a major stable QTL for TGW (*QTgw.cib-2A.2*) which co-localized with four QTLs for grain size traits (*QGl.cib-2A.3*, *QGa.cib-2A.3*, *QGd.cib-2A.1*, and *QGp.cib-2A.2*). ZKM138 also contributed to the beneficial alleles for all QTLs.

Additionally, three clusters associated with grain shape traits were located on chromosomes 2A (C2A.1), 4A (C4A), and 5A1 (C5A). C2A.1 consisted of three major stable QTLs (*QGl.cib-2A.1*, *QGr.cib-2A*, and *QLwr.cib-2A*), with ZKM138-derived alleles decreasing GL and LWR but increasing GR. Both C4A and C5A contained QTLs for GR, LWR, and GL, indicating that these three loci might control grain shape mainly by moderating grain length.

### Quantitative trait loci × environment effect evaluation based on the multi-environment trials module

Using the MET module under multiple environmental conditions, a total of four hundred QTLs for TGW and GRTs were detected (Supplementary Table 5). Consistent with Cui et al. (2014) and Fan et al. (2019), all QTLs detected by the BIP module could be repeatedly identified in the MET module, indicating that the QTLs detected in this study have high reliability and consistency. Among them, fifty-five QTLs were detected in a single environment, indicating that they are greatly affected by the environment and cannot be stably expressed. For example, *QGd.cib-6D1.2*, one of the environmentally sensitive QTLs with a high PVE (AbyE) of 6.15% (Supplementary Table 4), was specifically expressed in E2 (Supplementary Table 4). However, the PVE (AbyE) of *QTgw.cib-6A* was only 1.23%, while its PVE (A) was higher (7.04%) (Supplementary Table 5), indicating that this locus is relatively stable in different environments and mainly affected by genetic factors.

### Identification of known genes

The polymorphism between the two parents was tested by sixteen functional markers related to fourteen genes for grain weight and yield (Supplementary Table 6). Only *TaGS* (Zhang et al., 2014) and *TaTGW6* (Hanif et al., 2016) presented polymorphism between parents, but they did not overlap with any of the QTLs detected in this study (Supplementary Figure 4A).

Additionally, based on the detection of markers reported in previous studies, no divergence was found between the parents and lines for *TaGW2* and *TaBT1* (Supplementary Figure 4B). The sequencing of the promoter region and coding region of *TaGW2* further showed that they were consistent between parents (Supplementary Figures 4C,D). Therefore, *QTgw.cib-6A* was deduced to probably be a novel locus that differed from its nearby *TaGW2* and *TaBT1*. Therefore, *QTgw.cib-6A* was deduced to be a novel locus that differed from its nearby *TaGW2* and *TaBT1*.

### Pyramiding effects of three stable quantitative trait loci on thousand grain weight

**FIGURE 3:**
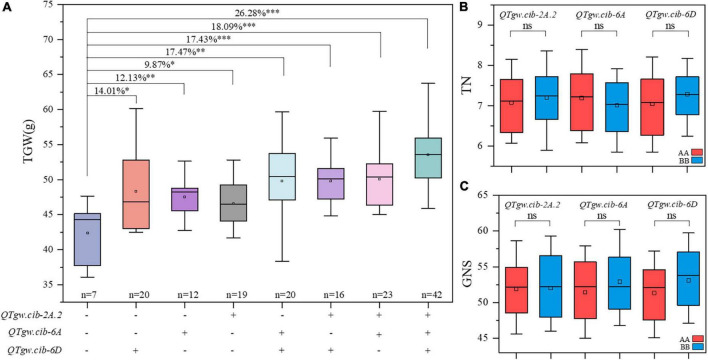
Pyramiding effect analysis of *QTgw.cib-2A.2*, *QTgw.cib-6A* and *QTgw.cib-6D* on TGW **(A)**, TN **(B)**, and GNS **(C)**. *, ^**^, and ^***^ represent significance at *P* < 0.05, *P* < 0.01, and *P* < 0.001, respectively and ns represents non-significance; + and - represent lines with and without the positive alleles of the target QTLs based on the flanking markers of the corresponding QTL, respectively; AA and BB represent lines with and without the positive alleles of the target QTLs based on the flanking markers of the corresponding QTL, respectively.

The additive effects of the positive alleles at the three QTLs for TGW (*QTgw.cib-2A.2*, *QTgw.cib-6A*, and *QTgw.cib-6D*) were analyzed (Figure 3A). Compared with those without favorable alleles, RILs carrying one, two, or three alleles showed TGW significantly increased TGW by 9.87–26.28%. The combination of the three superior alleles had the largest pyramiding effect on increasing TGW, followed by two superior alleles, and only one superior allele exhibited the smallest positive effect on TGW. This is further supported by line BC-168 carrying the ZKM138-derived allele at the three loci displaying a higher TGW than line BC-161 carrying the CM44-derived allele (Figure 1A). Moreover, these QTLs had no significant trade-off effect on TN (Figure 3B) and GNS (Figure 3C).

### Narrowing the *QTgw.cib-6A* interval using the wheat 50K single nucleotide polymorphism array and bulked segregant analysis with RNA sequencing

**FIGURE 4:**
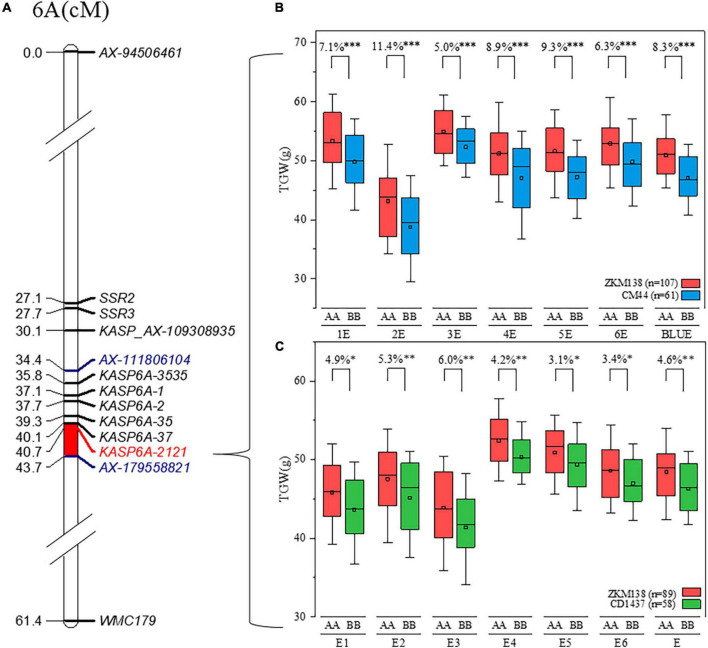
Delimitation and validation of *QTgw.cib-6A*. **(A)** The new integrated genetic map of *QTgw.cib-6A*; markers in blue represent the preliminary QTL interval of *QTgw.cib-6A*; markers in red represent tightly linked marker; and the bar in red represents the new interval of *QTgw.cib-6A* based on the integrated genetic map. **(B)** Effect estimation of *QTgw.cib-6A* on TGW in the BC population; the red and blue boxes indicate the TGW (g) performance of the lines with alleles from ZKM138 and CM44, respectively; *, ^**^, and ^**^ represent significance at *P* < 0.05, *P* < 0.01, and *P* < 0.001, respectively. **(C)** Effect estimation of *QTgw.cib-6A* on TGW in the ZC population. The red and green boxes indicate the TGW (g) performance of the lines with alleles from ZKM138 and CD1437, respectively; *, ^**^, and ^***^ represent significance at *P* < 0.05, *P* < 0.01, and *P* < 0.001, respectively.

Using the BSR-Seq approach, a set of candidate SNPs (70.43%) between the parents putatively associated with TGW were identified on chromosome 6A (Figure 2D), consistent with the QTL mapping results (Figure 2B). Combining the information from the candidate SNPs of BSR-Seq and the Wheat 50K SNP array, a new integrated genetic map for the candidate region of *QTgw.cib-6A* was reconstructed with thirty-nine KASP markers and seven SSR markers (Supplementary Table 7). After remapping based on the new map, the original candidate region (101 Mb) was narrowed to only 4.1 Mb (Figure 4).

The effects of *QTgw.cib-6A* on TGW and GRTs were further examined using the newly developed and linked marker (*KASP6A-2121*) in all datasets. Significant differences (*P* < 0.001) were observed in TGW (Figure 4) and all five grain size traits (GA, GD, GL, GW, and GP) (Supplementary Figure 5) between the lines with homozygous alleles from ZKM138 and CM44. However, no significant differences were found in grain shape traits, such as GR and LWR, at this locus (Supplementary Figure 5).

### Verification of *QTgw.cib-6A* in different genetic backgrounds and its geographic patterns

Considering that the favorable allele of *QTgw.cib-6A* was from ZKM138, the ZC-RILs also derived from the common parent ZKM138 were used to preliminarily validate the effects of *QTgw.cib-6A* under different genetic backgrounds. The lines containing different alleles showed significant differences in TGW (*P* < 0.05) (Figure 4). Moreover, the lines with the ZKM138-derived allele had significantly (*P* < 0.05) higher GW, GA, GP, and GD than those homozygous for the CD1437 allele (Supplementary Figure 5). To a certain extent, it was confirmed that the ZKM138-derived allele at this locus improves grain weight and most grain size traits in ZC-RILs.

**FIGURE 5:**
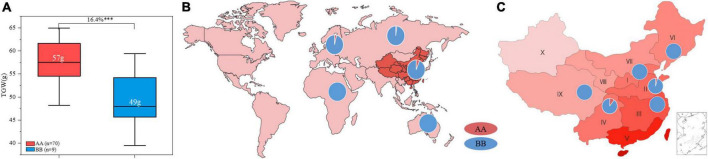
Transmissibility and geographic patterns of the genotype of *QTgw.cib-6A*. (**A**) Contributions of *QTgw.cib-6A* to TGW enhancement in 79 ZKM138-derivatives. Numbers in white represent the mean value of TGW; ^***^ represents significance at *P* < 0.001. Geographic distributions of the superior allele of *QTgw.cib-6A* in 624 worldwide samples **(B)** and 433 Chinese varieties **(C)**. I, Northern Winter Wheat Zone; II, Yellow and Huai River Valleys Facultative Wheat Zone; III, Middle and Lower Yangtze Valleys Autumn-Sown Spring Wheat Zone; IV, Southwestern Autumn-Sown Spring Wheat Zone; V, Southern Autumn-Sown Spring Wheat Zone; VI, Northeastern Spring Wheat Zone; VII, Northern Spring Wheat Zone; VIII, Northwestern Spring Wheat Zone; IX, Qinghai-Tibetan Plateau Spring-Winter Wheat Zone; X, Xinjiang Winter-Spring Wheat Zone; AA and BB represent varieties with and without the superior allele of *QTgw.cib-6A*, respectively.

ZKM138 has been an important backbone parent during our breeding process and derived several varieties and stable lines. In this study, a total of seventy-nine ZKM138 derivatives were used to further validate the effect of *QTgw.cib-6A* and estimate its transmissibility. In total, 88.6% of these lines harbored a ZKM138-derived allele, indicating that this candidate gene had been selected during the small-scale breeding process that used ZKM138 as the major parent (Figure 5A). The group harboring the superior alleles also exhibited significantly (*P* < 0.001) increased TGW (16.4%), which further confirmed the contribution of the ZKM138-derived allele to effective increases in TGW (Figure 5A).

To determine whether the superior allele was applied to a broader breeding range, using marker *KASP6A-2121*, we preliminarily investigated the geographic distribution of this genotype in 433 Chinese wheat varieties and 191 germplasms varieties from other fifteen countries belonging to Asian, Europe, Oceania, and Africa, respectively. As shown in Figure 5, the samples with AA genotype in 624 worldwide varieties (consistent with ZKM138) accounted for only 4.97% (31/624), of which 5.22% (29/556) was from Asian and 4% (2/50) was from Europe. No samples contained superior allele in Oceania and Africa. Notably, the ZKM138-derived allele was detected in 28 of 433 (6.47%) Chinese varieties, which were derived from wheat zones I, II, III, IV, VI, and IX. The frequency of favorable allele in Chinese wheat zones was in the order of IV (11.32%) > II (3.92%) > I, III, VI, and IX (0%) (Figures 5B,C and Supplementary Table 8). These results suggested that the superior allele could be used more widely as an insufficiently selected and applied locus to increase yield potential in the future.

### Candidate gene prediction of *QTgw.cib-6A*

**FIGURE 6:**
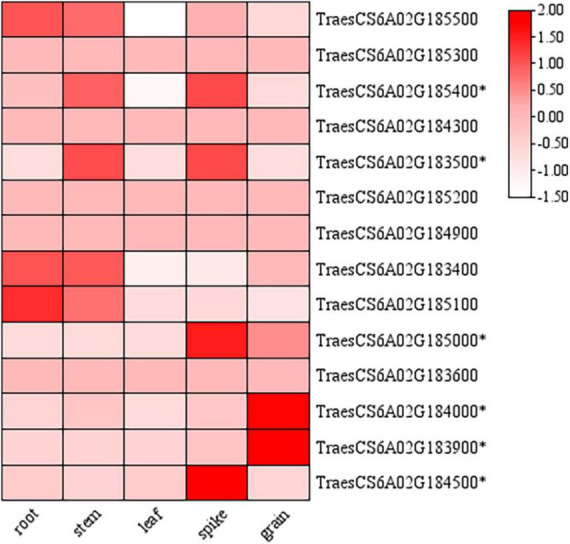
Expression patterns of candidate genes in the physical interval of *QTgw.cib-6A* in different tissues. * represents genes specifically expressed in the spike and grain; the data of expression profiles were obtained from the public online database (http://202.194.139.32/expression/wheat.html).

According to the re-sequencing data of the parents, fourteen genes in the candidate region of *QTgw.cib-6A* (213.1-217.2 Mb) had different SNPs in the gene body of the parents. These genes were annotated based on the Ensembl Plant database^6^ (Supplementary Table 9). The initial spatial expression pattern analysis showed that nine genes were expressed in various tissues (root, stem, leaf, spike, and grain) based on the public expression database^7^ (Borrill et al., 2016; Ramírez-González et al., 2018; Figure 6). Only six of them (*TraesCS6A02G185400*, *TraesCS6A02G183500*, *TraesCS6A02G185000*, *TraesCS6A02G184000*, *TraesCS6A02 G183900*, and *TraesCS6A02G184500*) were specifically expressed in the spike and grain, and might be the most likely candidate genes needing further validation.

## Discussion

### Thousand grain weight and grain shape are probably genetically independent in the BC-RILs genetic background

Grain shape and size traits are two kinds of grain features affecting the cereal milling process and yield formation, respectively (Peng et al., 2003). In this study, consistent with previous reports (Cheng et al., 2017; Wang et al., 2019a), wheat grain size traits showed a higher positive correlation coefficient with TGW (*r* > 0.67) than grain shape traits (*r* < 0.24), thus facilitating final yield, which could be partially explained by 12 co-localized QTLs (Figure 1B and Supplementary Table 4). These QTLs all presented the same direction of additive effects, while no stable QTLs affecting both TGW and grain shape traits were identified in this study, further indicating that modifying grain size might be an effective method to increase grain weight and that grain shape is more likely to be independent of TGW.

Unlike in the rice breeding process, where grain shape is under strong selection because of its contribution to rice processing and cooking quality (such as milling rate) (Jain et al., 2004; Zheng et al., 2010), grain shape does not appear to be a major target in the wheat selection and breeding process (Kovach et al., 2007). In fact, previous studies found that the flour extraction rate was higher for semispherical grains (62.1%) than for long grains (57.4%) in wheat (Cheng et al., 2020), indicating the equal importance of grain shape and grain size for wheat grain improvement. Thus, as shown in this study, pleiotropic QTLs for grain size and TGW could be used to enhance grain yield, and two major stable QTLs (*QGr.cib-2A* and *QLwr.cib-2A*) for grain shape might also play a role of in process quality improvement.

### Comparison of quantitative trait locus with previous studies

Identifying beneficial alleles has been an effective method for wheat genomic research (Chen et al., 2017). In this study, there were significant differences between parents for TGW and other grain traits (Figure 1A). ZKM138 was found to present superior genotypes at only three genes (Supplementary Table 6), including *TaTGW6* (Hanif et al., 2016), *TaTGW7* (Hu et al., 2016), *TaSus1-7A* (Hou et al., 2014), and carry negative genotypes at all of the other ten genes (Supplementary Table 6), suggesting the possibility of the undiscovered loci supporting high TGW performance in ZKM138. In this study, eight QTL clusters containing stable QTLs for TGW and GRTs were detected, but most of them were previously reported, except C6A harboring *QTgw.cib-6A*. Specifically, the TGW relative gene, *TaFlo2-A1*, located on deletion bin “2AL1–0.85–1.00” (Sajjad et al., 2017), was overlapped with the CI of C2A.3, which was consistent with numerous reported QTLs for TGW (Ma et al., 2018; Sukumaran et al., 2018; Liu et al., 2019), and thus may be responsible for this region affecting TGW. And another gene associated with TGW, *TaGS1a* (Guo et al., 2013), was located in the region of C6D1.2 and thus might be the gene responsible for this QTL cluster.

At the QTL level, the CI of C2A.1, close to *Ppd-A1* (a well-known photoperiod locus also affecting GRTs) (Beales et al., 2007), was overlapped with several reported QTLs for TGW (Mohler et al., 2016; Liu et al., 2019). Additionally, QTLs for TGW and grain size traits previously detected in this region by Mangini et al. (2021) in durum wheat. However, considering that this region was mainly associated with grain shape and did not affect TGW, differences between C2A.1 and the above reported QTLs were possible. Another cluster mainly controlling grain shape, C5A1, was located in a 12.5 cM interval corresponding to 0.64–3.65 Mb. In this region, only one QTL for GL was reported at 2.79 Mb (Sun et al., 2017), which might correspond to C5A1, which also harbored a stable QTL for GL (*QGl.cib-5A1*) (Table 2).

For grain size related loci, the QTL cluster C2A.2, associated with TGW and four grain size traits (GL, GA, GD, and GP), might include QTLs for TGW and grain size traits that have been previously reported in hexaploid wheat (Cui et al., 2014; Guan et al., 2018; Li et al., 2022) and in durum wheat (Peleg et al., 2011; Sun et al., 2020). Moreover, the region of C6D1.1 contained QTLs for four grain size traits that were consistent with these previously reported QTLs for TGW (Chen et al., 2019; Liu et al., 2019), GL, and GW (Chen et al., 2019).

### Three genomic regions exhibit an inappreciable trade-off

Wheat yield is a polygenic trait primarily determined by three yield components, i.e., TGW, GNS, and TN (Simmonds et al., 2016). Commonly, TGW is negatively associated with GNS (Jia et al., 2013; Schulthess et al., 2017; Zhai et al., 2018), and thus, many TGW-related genes present a trade-off effect on grain yield (Sukumaran et al., 2018). For example, Zhai et al. (2018) reported a novel and rare *TaGW2-A1* allele that increased TGW by reducing GNS. Identifying QTLs for TGW with no negative effect on GNS is beneficial for enhancing yield potential. However, at the QTL level, Xie and Sparkes (2021) reported that 96% of MQTL had a trade-off between TGW and GNS. In this study, three stable QTLs for TGW (*QTgw.cib-2A.2*, *QTgw.cib-6A*, and *QTgw.cib-6D1*) with superior alleles from ZKM138 were detected. The combination of two or three of them showed a significant additive effect on TGW and had no noticeable negative effect on GNS (Figure 3), suggesting that they have potential value for wheat breeding. Similar results were reported by Griffiths et al. (2015) and Golan et al. (2019). Golan et al. (2019) found a unique *GNI-A1* allele in wild emmer that promoted grain weight without significantly affecting grain number, contributing to breeding efforts to enhance grain yield.

### Potential breeding values of *QTgw.cib-6A*

Linked maker is useful for conveniently detecting target loci (Collard and Mackill, 2008). In this study, a major stable QTL *QTgw.cib-6A* for TGW and grain size was mapped and finally narrowed to a 213.1-217.2 Mb region (∼4.1 Mb) on chromosome 6A with a tightly linked KASP marker (*KASP6A-2121*) (Figure 4). In previous studies, linked markers were used to investigate the transmissibility (Fan et al., 2018) and distribution (Liu et al., 2020) of a particular genotype of a target QTL. These reports found that many favorable alleles or genotypes have been widely used in genetic improvement (Chen et al., 2022; Deng et al., 2022). For instance, the detection of functional markers of *FT-D1* (Chen et al., 2022) and *TaGW2* (Su et al., 2011) revealed that their superior haplotypes had been frequently selected among Chinese varieties. For instance, the frequency of the positive allele of *TaGW2* has gradually increased (from 58.3 to 77.4%) in recent decades. Additionally, the excellent allele of *FT-D1* occurs at a high frequency (more than 60%) in different worldwide wheat regions (Chen et al., 2022). The high frequency and range of applications of these well-known genes indicate their large contributions to yield improvement in the artificial breeding process. On the other hand, a possible novel gene with a relatively low frequency distribution might provide a new target for genetic improvement and an extra opportunity for further yield enhancement.

In this study, among the 79 derivatives of ZKM138, the strong artificial selection of the ZKM138 genotype representing high TGW was observed (88.6%), possibly because its effect on increasing yield has been passively noticed and selected during the breeding process, mainly with ZKM138 used as a parent. In addition, only 4.97% (31/624) of the worldwide varieties carried superior genotypes increasing TGW, with the highest frequency 11.32% (18/159) in Southwestern Wheat Zone in China, suggesting that this locus more likely harbored a new and underutilized gene controlling TGW with potential application value in the subsequent high-yield variety breeding process.

## Conclusion

In this study, we performed QTL analysis using seven datasets from the BC-RILs *via* a new genetic map based on the Wheat 50K SNP array. A total of ninety-one QTLs for TGW and GRTs were detected on fourteen chromosomes. Among them, thirty-six stable QTLs were identified in more than two datasets, and eight genomic clusters were highlighted. The pyramiding of three of the QTLs (*QTgw.cib-2A.2*, *QTgw.cib-6A*, and *QTgw.cib-6D1*) for TGW was found to contribute greatly to high wheat yield, which might primarily support the excellent grain weight and yield performance of ZKM138. Notably, *QTgw.cib-6A* was identified in all datasets and finally delimitated to a physical interval of approximately 4.1 Mb. Further transmissibility and geographic pattern analysis revealed that the superior allele was selected during our actual breeding process based on ZKM138 but is still rare in worldwide modern wheat varieties. Thus, the superior allele of *QTgw.cib-6A* and its linked markers might be valuable for further improving TGW and yield.

## Data availability statement

The data presented in the study are deposited in the NCBI repository, accession number: PRJNA850613.

## Author contributions

XL undertook the field trials and subsequent analysis of all available data including the phenotyping and population genotyping and drafted this manuscript. ZX assisted in field trials. BF, QZ, GJ, SL, SG, and DL participated in phenotyping. ZX, XF, and TW developed the BC population. XL, XF, ZX, and TW discussed the results. XF and TW designed the experiments, guided the entire study, participated in data analysis, discussed the results, and revised the manuscript. All authors contributed to the article and approved the submitted version.

## Footnotes

1
http://faostat.fao.org

2
http://www.fao.org

3
https://wheatgenome.org/

4
http://www.isbreeding.net/

5
http://202.194.139.32/expression/wheat.html

6
http://plants.ensembl.org/index.html

7
http://202.194.139.32/expression/wheat.html
