# Supplementary material for: Quantitative trait loci identification and breeding value estimation of grain weight-related traits based on a new wheat 50K single nucleotide polymorphism array-derived genetic map
Source: Front Plant Sci. 2022 Aug 30;13:967432. doi: 10.3389/fpls.2022.967432 (PMC9468616; doi:10.3389/fpls.2022.967432)
Supplement: Supplementary file 2 [file Data_Sheet_2.docx]

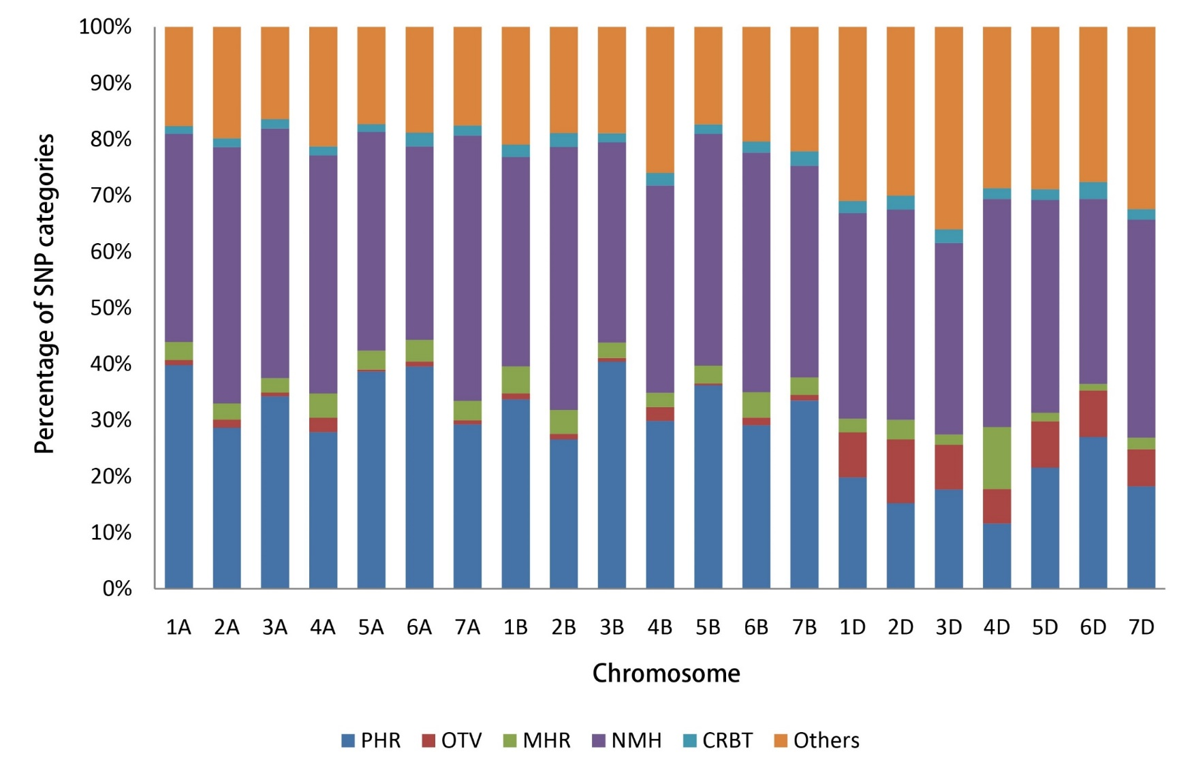


**Supplementary Figure 1** Categories of SNP markers for genetic map construction. PHR, Poly High Resolution; MHR, Mono High Resolution; NMH, No Minor Hom; CRBT, Call Rate Below Threshold.
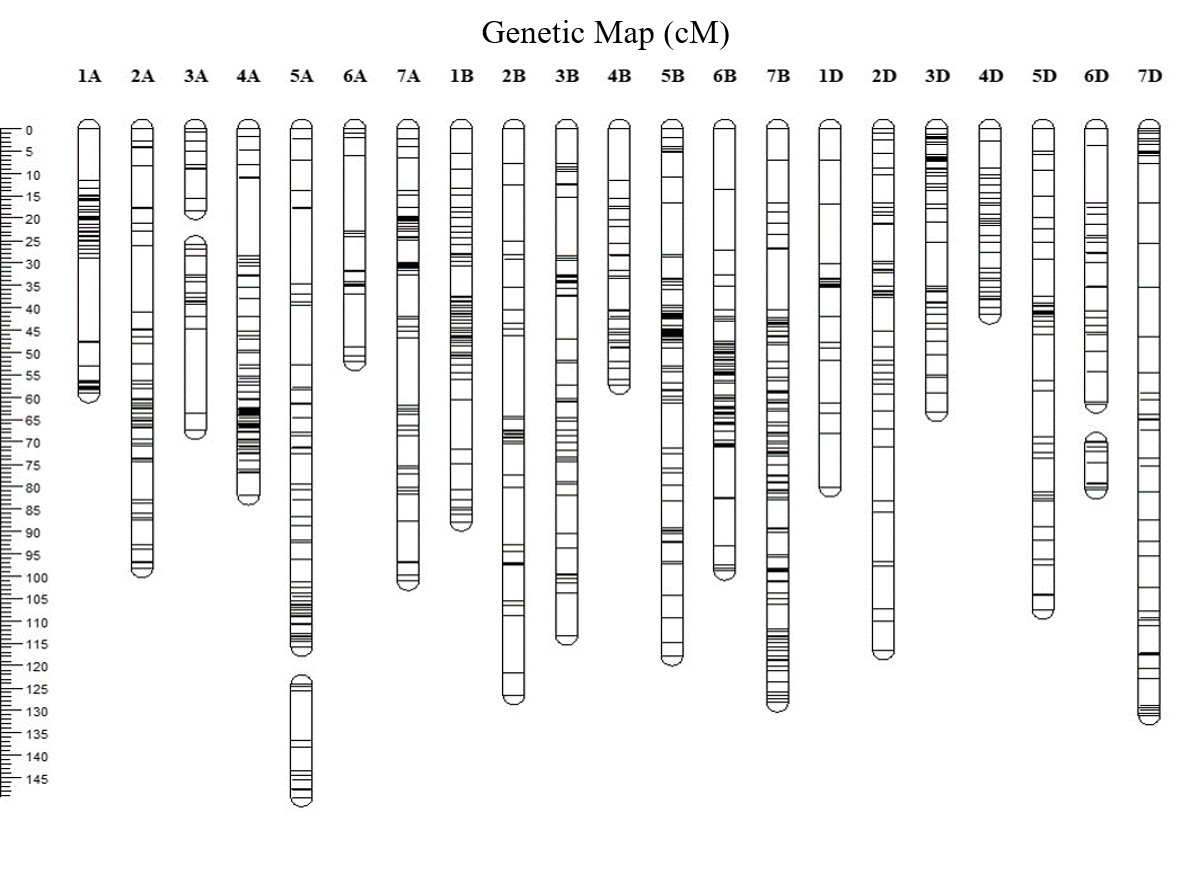


**Supplementary Figure 2** A new genetic map of the BC-RILs based on the Wheat 50K SNP array. Only one bin marker is shown in this figure, and the names and positions of all markers are listed in Supplementary Table 3.
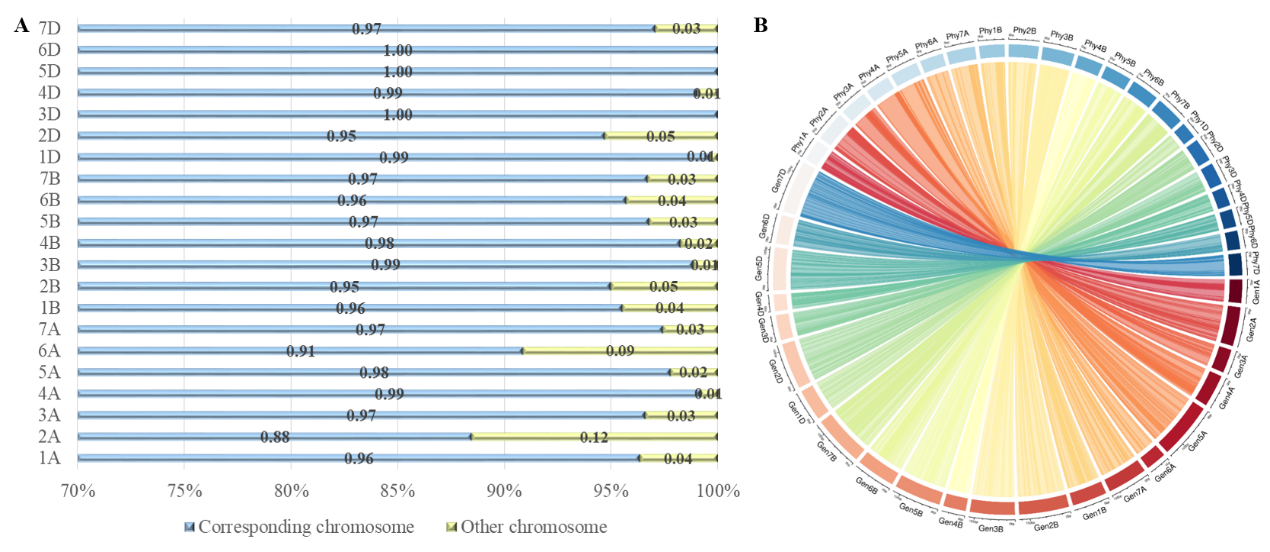


**Supplementary Figure 3** Colinearity between genetic maps and physical reference maps. Gen1A to Gen7D represent the 21 wheat chromosomal genetic maps released in this paper; Phy1A to Phy7D represent the 21 wheat chromosomal physical maps.
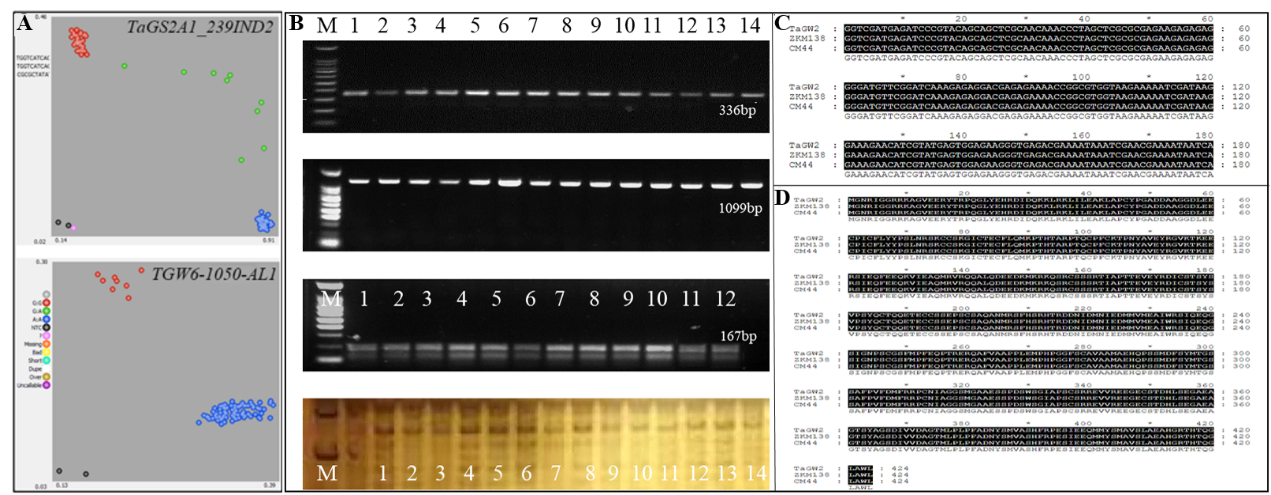


**Supplementary Figure 4** Identification of known genes. (**A**) Identification of polymorphisms via PCR products amplified with primers. Lanes 1–4: ZKM138, CM44, ZKM138, and CD1437; lanes 5–9: lines in the BC-RILs; lanes 10–14: lines in ZC-RILs; M: DNA ladder. (**B**, **C**) Comparative analysis of sequencing results in promoter and coding region of parents. (**D**) Fluorescence PCR typing results of KASP markers *TaGS2A1_239IND2* and *TGW6-1050-AL1* in the BC population.
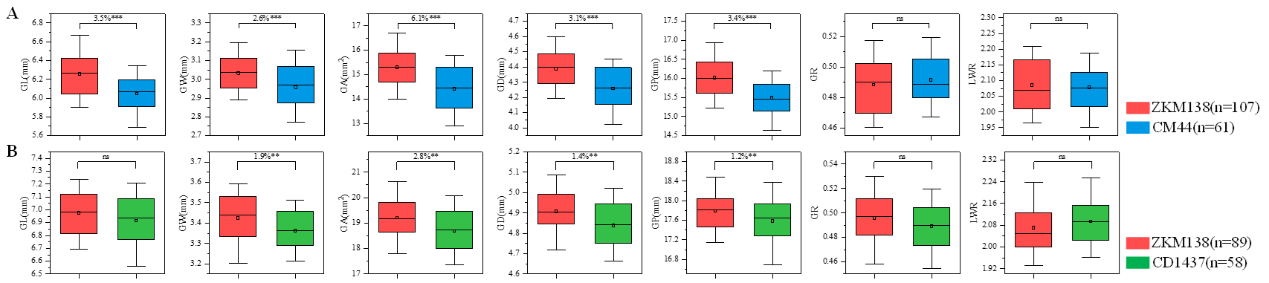


**Supplementary Figure 5** Additive effects of *QTgw.cib-6A* on GL (mm), GW (mm), GA (mm^2^), GD (mm), GP (mm), GR, and LWR in the BC-RILs (**A**) and ZC-RILs (**B**). The red, blue, and green boxes represent the performance of these grain related traits with alleles from ZKM138, CM44 and CD1437, respectively; *, ** and *** indicate significance at *P* < 0.05, *P* < 0.01 and *P* < 0.001, respectively.
